# Supplementary material for: Identification of novel DNA sequence motifs that modulate transcription in T cells
Source: BMC Genomics. 2026 Jan 9;27:154. doi: 10.1186/s12864-025-12425-9 (PMC12879379; doi:10.1186/s12864-025-12425-9)
Supplement: Supplementary file 1 — Supplementary Material 1. [file 12864_2025_12425_MOESM1_ESM.docx]

**Supplemental Figures and Tables**

**Supplemental Figure S1**. **Number of RNA-seq samples used for gene expression analyses.** RNA-seq data from the DICE consortium (12) and the SRA (see Supplemental Table S1) were used to identify protein-coding genes expressed in T cells. The plot displays the number of RNA-seq samples used to calculate the median expression for each protein-coding gene in the genome across each cell type independently (y-axis).

**Bioproject accessions for the RNA-seq datasets obtained from the SRA.**

| **Cell Group** | **Study** | **Cell Type** | **Number of Samples** |
| --- | --- | --- | --- |
| T cell | PRJNA494278 | Naive CD4^+^ T cells | 101 |
|  |  | Naive CD8^+^ T cells | 102 |
|  |  | Follicular Helper T cells | 104 |
|  |  | Th1 | 95 |
|  |  | Th1-17 | 103 |
|  |  | Th17 | 104 |
|  |  | Th2 | 104 |
|  |  | Memory Regulatory T cells | 104 |
|  |  | Naive Regulatory T cells | 103 |
|  |  | Naive B cells | 106 |
|  |  | Classical Monocytes | 106 |
|  |  | Non-Classical Monocytes | 105 |
| Non-T cell | PRJNA687693 | Brain microvascular endothelial cells | 12 |
|  |  | Pulmonary endothelial cells | 12 |
|  | PRJNA515044 | Brain endothelial cells | 16 |
|  | PRJNA579487 | Aortic endothelial cells | 104 |
|  | PRJNA431399 | Terminally differentiated hepatic cells | 20 |
|  | PRJNA622787 | Hepatocyte cell line | 36 |
|  | PRJNA726552 | Sperm cells | 6 |
|  | PRJNA310976 |  | 34 |
|  | PRJNA666614 | Oocyte cells | 75 |
|  | PRJNA484890 |  | 20 |
|  | PRJNA211925 | CD34^+^ CD45RA^-^ hematopoietic stem cells from cord blood | 136 |
|  | PRJNA419347 | CD34^+^ peripheral hematopoietic stem cells | 32 |

**Supplemental Table S1.** PRJNA494278 was obtained from the dbGaP website, under phs001703.v1.p1 (Project #21785). The table includes the cell type(s) for each dataset and the number of samples used.


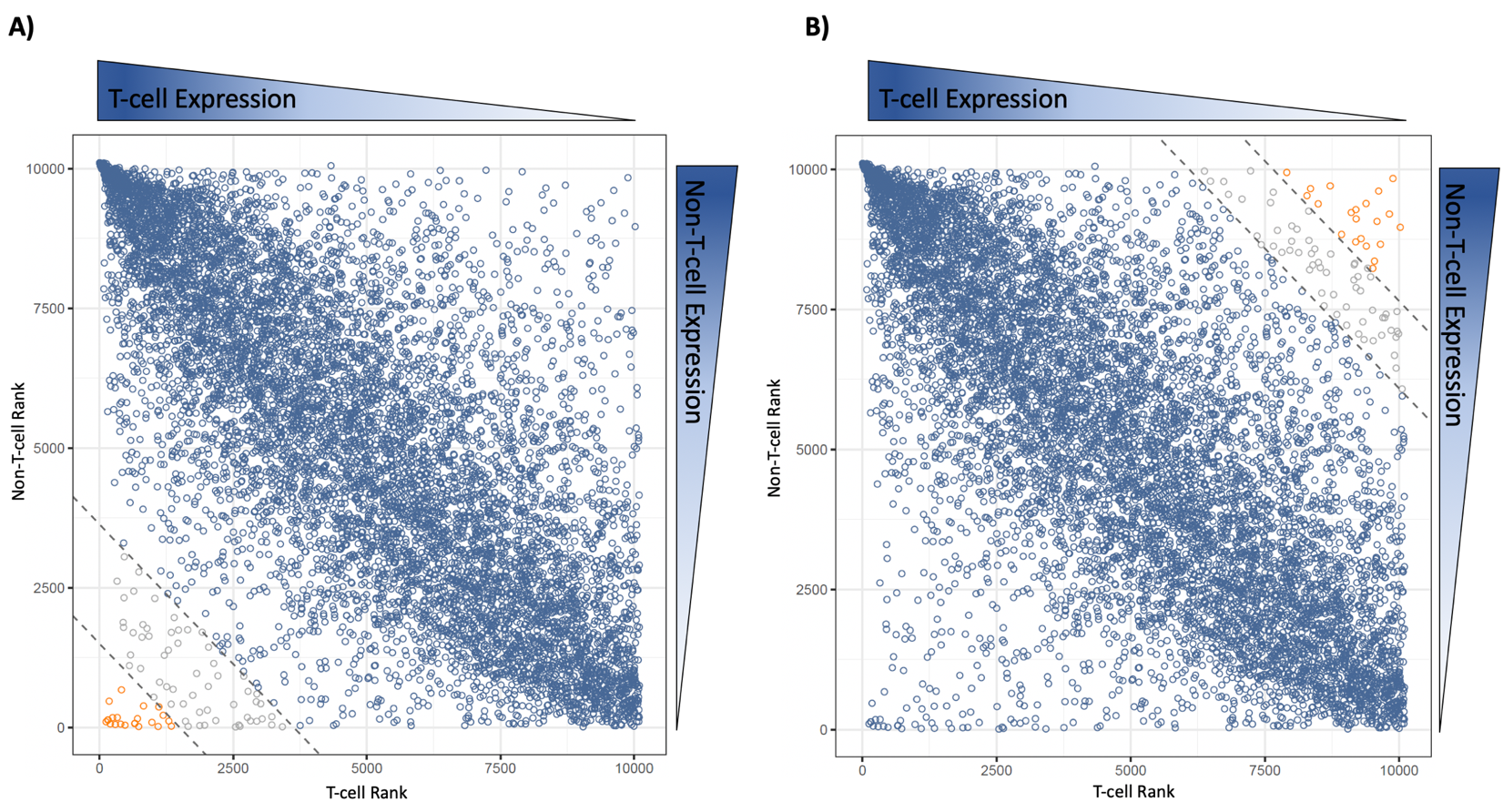
**Supplemental Figure S2. The relationship between gene ranks for T-cells and non-T-cells.** The plots display the ranks for all genes expressed in T cells. Genes with higher expression in T cells receive a higher T-cell rank (x-axis), and genes with lower expression in non-T cells receive a higher non-T-cell rank (y-axis). A) The top-ranked T-cell genes (n=22, shown in orange) have a specificity score that is more than four standard deviations from the mean. The comparator gene set containing the non-specific T-cell genes (n=8,562, shown in blue) have specificity scores within three standard deviations from the mean. B) We created a size-matched control gene set by selecting the 22 bottom-ranked genes as our genes of interest (orange) and the top 8,562 ranked T-cell genes as our comparator set of genes (blue).


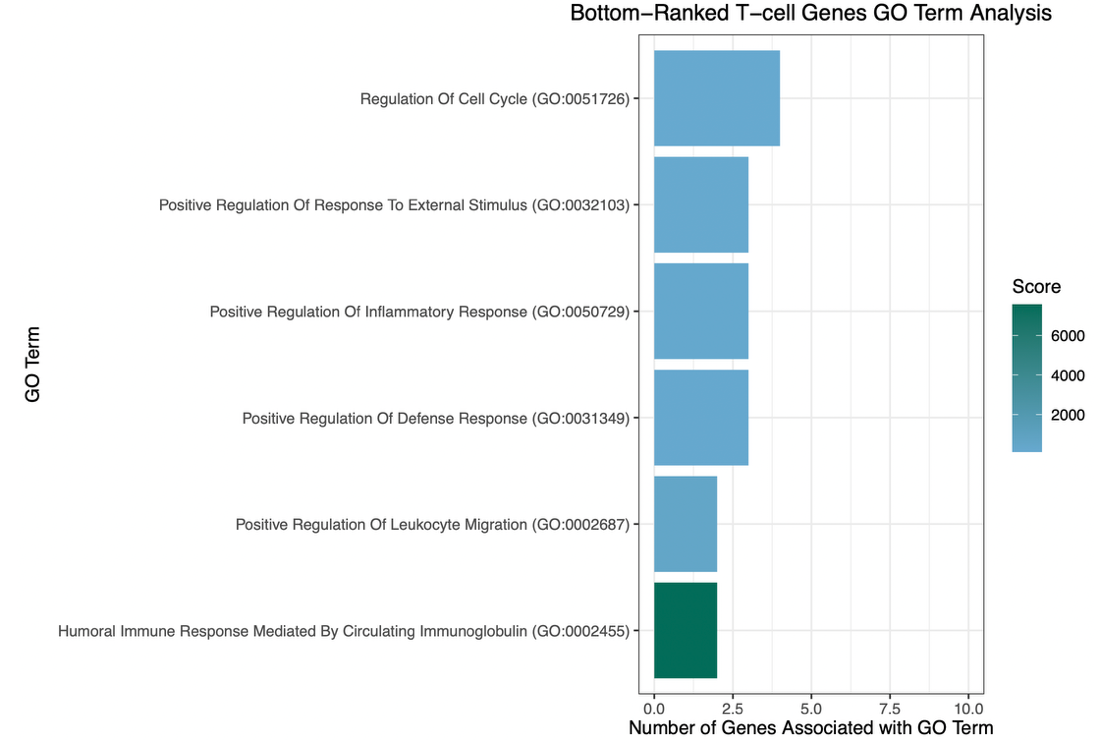


**Supplemental Figure S3. The 22 bottom-ranked T-cell genes are associated with immune-related biological processes.** The bar plot shows the enriched GO terms (y-axis, Benjamini-Hochberg corrected, p-value < 0.05) for the 22 bottom-ranked T-cell genes. The bars correspond to the number of genes associated with the specified GO Term. Colour corresponds to the score obtained from the GO term analysis. GO terms are ordered by their adjusted p-value.

**Supplemental Figure S4. Illustration of the empirical framework used to identify enriched motifs.** The framework utilizes a set of genes of interest (top-ranked T-cell genes) and a set of comparator genes (non-specific T-cell genes). For the genes of interest, the OCRs are obtained, and FIMO is used to identify sites within the OCRs corresponding to TFBS motifs or novel motifs. The framework then obtains 10,000 sets of genes of the same length as the genes of interest and repeats the process. Motifs are enriched if less than 5% of the random samplings exhibited a level of enrichment as large as the level observed for the genes of interest. Additionally, there needs to be at least twice as many genes of interest with the motif compared to the comparator set of genes.

**Supplemental Figure S5.** The statistical framework was used to identify enriched novel motifs in distal (pink) and proximal (blue) regulatory regions. Non-enriched motifs are shown in grey. FIMO was used to identify sites for all novel motifs in regulatory regions for the 22 top-ranked T-cell genes (y-axis). The regulatory regions for the 22 top-ranked T-cell genes were randomly repositioned across the genome 10,000 times to obtain the expected number of motif sites in the genome (x-axis).

**Supplemental Figure S6. Candidate novel motifs and TFBS selected for validation using the STARR-seq assay.** A) Novel motifs and B) TFBS were selected for their enrichment in regulatory regions for the top-ranked T-cell genes (see Methods).

**Supplemental Figure S7. Flow Cytometry results for the positive control and synthetic background sequence.** Jurkat and K562 cells were transfected with the STARR-seq vector containing the synthetic background DNA sequence, or the positive control sequence (seq1305), alongside a vector containing mCherry as a transfection control. Transfections were successful for both cell lines, and GFP expression was detected using flow cytometry. A) Gating strategy used in the flow cytometry experiment. B) The synthetic DNA sequence designed *in silico* had minimal expression in Jurkat cells. The positive control sequence drove GFP expression at higher levels than the synthetic background sequence in both C) K562 and D) Jurkat cells.

**Information for the positive control and synthetic background DNA sequence used in the STARR-seq experiment.**

| **Sequence Name** | **Coordinates (hg38)** | **DNA Sequence** |
| --- | --- | --- |
| Seq1305 (strong enhancer) | chr1:61048980-61049180 (+) | CAGATGTGAGCCACAGCACCCGGCCTACAGCCGTTGTCTATGAGGAGATAAGCTAGTTATCAAAACTCCCCATAACCCATCCTGTTTCCTACTCAAACACTGAAACCTCTTAGCTCTCTCCCACTTCCTCTAAGGCCCTAATCTTCAAGGAACAGAAAGAAGCTAAACAGTCTTACAAGTTAGATTTCTTAGAATAAAAG |
| Synthetic Background Sequence | NA | TGACATCACATTAAGGGCCGATAGCATCCACTACCGCTTAAGCCGCACTAAAATGATAACATCATATCGTATTCGTAGTAACATCTACAGATTGCAGTAGTAATCGAGGTTTTGGGCTAGTTGGA |

**Supplemental Table S2.** The positive control DNA sequence was previously validated in K562s using a Lenti-MPRA experiment (32). The sequence was chosen due to a high activity score in K562, indicative of increased gene transcription. We also created a synthetic DNA sequence *in silico* to use as a background DNA sequence. The sequence was designed to have no TFBS or novel motifs in the sequence (see methods for details).

**Supplemental Figure S8.** Pearson correlation (r) between sequencing replicates (n=8) obtained from sequencing the STARR-seq plasmid library.


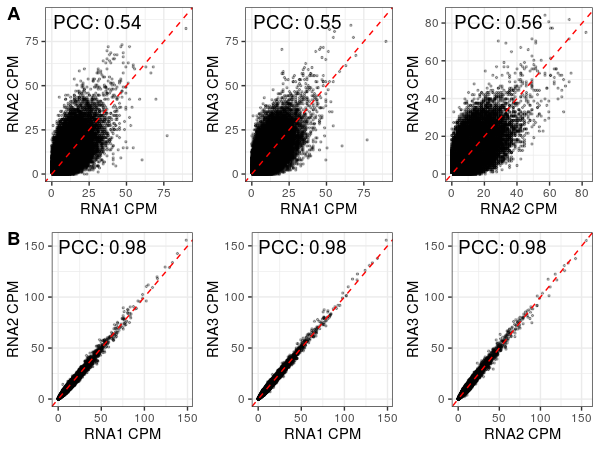


**Supplemental Figure S9.** Pearson correlations (r) for counts per million (CPM) derived from amplicon-sequencing replicates (n=3) for transfected A) Jurkat and B) K562 cell lines.


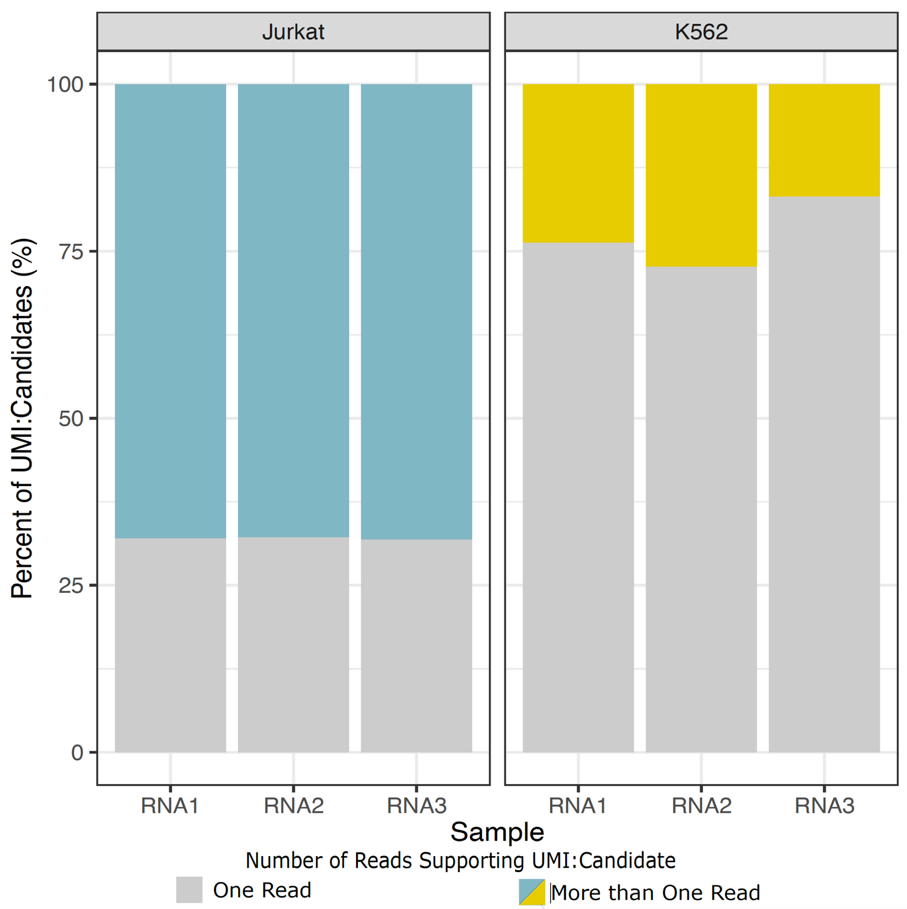


**Supplemental Figure S10.** The plot shows the number of distinct oligo-UMIs that are covered by more than one read in Jurkat and K562 cells across the three replicates. Jurkats had sufficient coverage, whereas K562 cells could have used additional amplicon sequencing.

**Supplemental Figure S11.** The positive control sequence (seq1305) drives transcription in Jurkat cells (left), as well as K562 cells (right). Most of the activity is driven by the 3’ end of the enhancer sequence.

**Supplemental Figure S12. The position of a motif relative to the core-promoter has a significant effect on gene transcription.** A) Schematic of the oligonucleotides used for the analysis. Only oligos containing a single copy of two distinct motifs were used to determine if the motifs of interest have a different effect on gene transcription depending on whether they are the first or second motif in the oligo relative to the core-promoter. B) The position of the motif relative to the core-promoter has a significant effect on gene transcription (* Wilcoxon t-test, Bonferroni corrected, p-value < 0.007) in B) Jurkat and C) K562 cells, left. The preference for the position is displayed in the figure on the right for both Jurkat and K562 cells.

**Supplemental Figure S13. Pairs of motifs have additive effects on gene transcription.** The activity scores for the pairs of motifs that have additive effects on gene transcription in Jurkats. Error bars represent the standard error.

**List of adapter and primer sequences used in the STARR-seq assay.**

| **Name** | **Sequence (5’ to 3’)** | **Purpose** |
| --- | --- | --- |
| **5’ Partial Illumina Adapter** | ACACTCTTTCCCTACACGACGCTCTTCCGATCT | Illumina Sequencing Adapters |
| **3’ Partial Illumina Adapter** | AGATCGGAAGAGCACACGTCTGAACTCCAGTCAC |  |
| **5’ Priming Site** | CTTCTATCTAGCACCGGT | Priming sites |
| **3’ Priming Site** | GTCGACGATGTAGGTACT |  |
| **SS_oligo_f** | CTTCTATCTAGCACCGGT | Amplifying the oligo library before cloning into the STARR-seq vector |
| **SS_oligo_r** | AGTACCTACATCGTCGAC |  |
| **SS_UMI_r** | AAGCAGAAGACGGCATACGAGAT**NNNNNNNNNN**GTGACTGGAGTTCAGACGTGT*G | First strand synthesis and integration of the 10bp UMI |
| **SS_intspan_f** | TCGTGAGGCACTGGGCAG*G*T*G*T*C | Junction PCR |
| **SS_P7seq_r primer** | CAAGCAGAAGACGGCATACGAGA*T | Junction PCR |
| **SS_i5_XX_f primer** | AATGATACGGCGACCACCGAGATCTACAC**NNNNNNNN**ACACTCTTTCCCTACACGACGCTCTTCCGATCT | Sequencing ready PCR to add the relevant Illumina sequencing adapters and i5 barcodes |
| **SS_P7seq_r primer** | CAAGCAGAAGACGGCATACGAGA*T |  |

**Supplemental Table S3. *** = phosphorothioate bond to protect against 3’-5’ exonuclease activity. **NNNNNNNNNN** = unique molecular identifier. **NNNNNNNN** = i5 barcodes. XX corresponds to the two-digit number corresponding to the i5/i7 primer pair UDI ID from Illumina (UDI00XX), where the i5 barcodes were obtained.

**Sequencing statistics for the DNA and amplicon libraries.**

| **Sample** | **Number of Paired-End Reads** | **Average Number of Paired-End Reads Per Oligo (n=59776)** |
| --- | --- | --- |
| **DNA1** | 20176782 | 337 |
| **DNA2** | 27200642 | 455 |
| **DNA3** | 20914051 | 349 |
| **DNA4** | 25458490 | 425 |
| **DNA5** | 26854834 | 449 |
| **DNA6** | 21413241 | 358 |
| **DNA7** | 23746091 | 397 |
| **DNA8** | 18430233 | 308 |
|  | | |
| **Jurkat RNA1** | 4362984 | 72 |
| **Jurkat RNA2** | 3479663 | 58 |
| **Jurkat RNA3** | 3153281 | 52 |
| **K562 RNA1** | 11631784 | 194 |
| **K562 RNA2** | 10436332 | 174 |
| **K562 RNA3** | 9776950 | 163 |

**Supplemental Table S4.** Sequencing statistics for the DNA and amplicon libraries.
